# The nucleobase analog 4-thiouracil hijacks the pyrimidine salvage pathway to inhibit *Staphylococcus aureus* growth

**DOI:** 10.1128/spectrum.00640-25

**Published:** 2025-05-27

**Authors:** Matthew J. Munneke, Jeffrey A. Freiberg, Eric P. Skaar

**Affiliations:** 1 Department of Pathology, Microbiology, and Immunology, Vanderbilt University Medical Center 204907, Nashville, Tennessee, USA; 2 Vanderbilt Institute for Infection, Immunology, and Inflammation, Vanderbilt University Medical Center 12328, Nashville, Tennessee, USA; 3 Division of Infectious Diseases, Department of Medicine, Vanderbilt University Medical Center 12328, Nashville, Tennessee, USA; University of Florida College of Dentistry, Gainesville, Florida, USA

**Keywords:** nucleotides, pyrimidine metabolism, *Staphylococcus aureus*, 4-thiouracil

## Abstract

*Staphylococcus aureus* is a leading cause of bacterial-induced mortality due to infections that are increasingly resistant to antibiotics, highlighting the need for new therapeutic strategies to treat these drug-resistant infections. Targeting essential pathways that differ from the host, such as cell wall synthesis, has served as an effective approach for antimicrobial drug development. Nucleotides are essential building blocks for nucleic acids and the bacterial cell wall, and we hypothesized that the metabolic pathways required to obtain these molecules may represent promising antimicrobial targets. To investigate if pyrimidine metabolism could be leveraged to inhibit *S. aureus* growth, we tested the antimicrobial activity of the uracil derivative, 4-thiouracil (4-TU). Growth of laboratory methicillin-susceptible and resistant strains, including a methicillin-resistant clinical isolate, is inhibited by 4-TU. Based on the structural similarity between 4-TU and uracil, we hypothesized that 4-TU hijacks the pyrimidine salvage pathway for incorporation into RNA. High-performance liquid chromatography (HPLC) analysis showed 4-thiouridine (s^4^U) in RNA isolated from *S. aureus* treated with 4-TU. Isolation of 4-TU-resistant *S. aureus* suppressor strains revealed that mutations in uracil phosphoribosyltransferase (Upp), a component of the pyrimidine salvage pathway, confer resistance to 4-TU. HPLC analysis of RNA isolated from an *S. aureus upp* mutant demonstrated a lack of s^4^U, suggesting that *upp* is required for incorporation of 4-TU into RNA. Expression of *Clostridioides difficile* thiouracil desulfurase in *S. aureus*, or cotreatment with uracil and 4-TU, alleviates 4-TU toxicity. Collectively, these findings suggest that 4-TU commandeers the pyrimidine salvage pathway to inhibit *S. aureus* growth.

**IMPORTANCE:**

*Staphylococcus aureus* is associated with greater than one million global deaths annually and is capable of infecting every human tissue. The increasing emergence of antibiotic-resistant strains emphasizes the urgent need to develop new therapeutic strategies to treat infections. Nucleoside analogs that disrupt pyrimidine or purine nucleotide metabolism serve as a promising approach for treating drug-resistant infections, as these pathways differ between host and bacteria. Here, we demonstrate that the uracil derivative 4-thiouracil (4-TU) inhibits *S. aureus* growth by hijacking the pyrimidine salvage pathway, leading to incorporation of 4-TU into RNA. We found that mutations in uracil phosphoribosyltransferase (*upp*) confer resistance to 4-TU and prevent incorporation into RNA. Expression of a thiouracil desulfurase (*tudS*) from *Clostridioides difficile* is sufficient to detoxify 4-TU and diminish 4-TU levels in RNA. Taken together, these results suggest that 4-TU-mediated disruption of pyrimidine metabolism limits *S. aureus* growth, which may serve as a promising therapeutic target.

## OBSERVATION

*Staphylococcus aureus* is an important human pathogen causing over half a million infections annually in the United States, including skin and soft tissue infections, endocarditis, osteomyelitis, pneumonia, and sepsis (1). To infect a broad array of vertebrate host environments, *S. aureus* employs strategies to acquire essential nutrients, including metals (2, 3), amino acids (4, 5), and nucleotides (6, 7). Due to the essentiality of metabolic adaptation to the host environment, targeting nutrient acquisition is an attractive strategy for new antimicrobial development (8). Transposon sequencing (TnSeq) screens of *S. aureus* have revealed genes essential for *S. aureus* fitness in multiple niches of the vertebrate host, with certain metabolic pathways demonstrating particular importance (5, 9, 10). Several genes predicted to be involved in purine and pyrimidine metabolism were found to be essential for fitness in abscesses and growth in *ex vivo* fluids (10). Furthermore, TnSeq analysis of *S. aureus* in a colony filter biofilm model revealed several genes involved in pyrimidine metabolism are important for biofilm growth (11). These findings highlight the importance of pyrimidine metabolism for *S. aureus* fitness in the abscess and during biofilm growth, motivating us to investigate this pathway to inhibit drug-resistant *S. aureus*.

**Fig 1:**
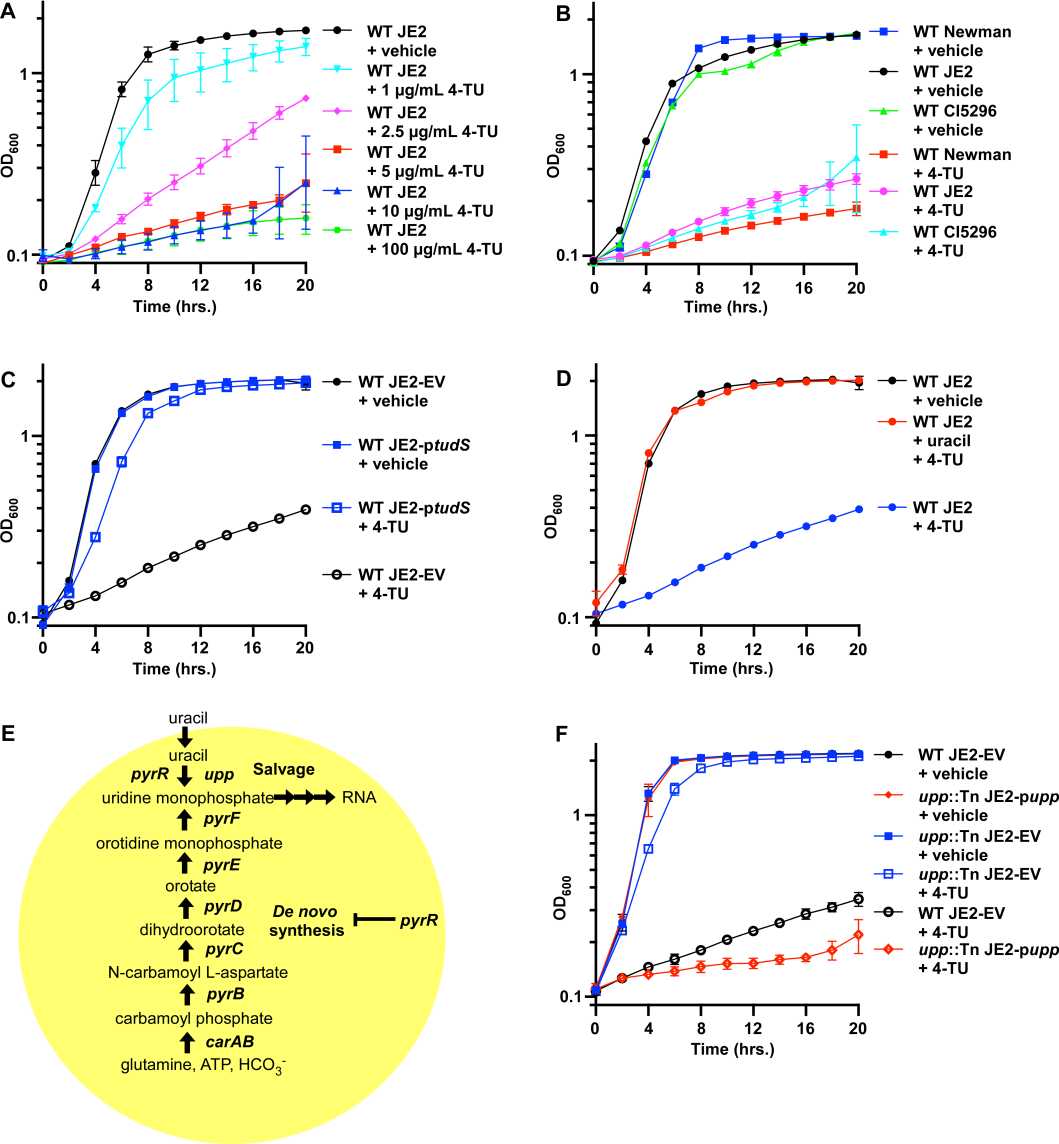
4-TU inhibits *S. aureus* growth through the pyrimidine salvage pathway. (**A**) Growth of wild-type (WT) *S. aureus* JE2 in dimethyl sulfoxide (vehicle) or in a range of concentrations of 4-TU in chemically defined media (CDM). (**B**) Growth of WT *S. aureus* Newman, JE2, and the Vanderbilt University Medical Center clinical isolate CI5296 treated with vehicle or 100 µg/mL 4-TU in CDM. (**C**) Growth of WT *S. aureus* JE2 harboring pOS1 empty vector (EV) or vector containing *C. difficile tudS* treated with vehicle or 100 µg/mL 4-TU. (**D**) Growth of WT *S. aureus* JE2 treated with vehicle, 100 µg/mL 4-TU, or 100 µg/mL 4-TU and 100 µg/mL uracil. (**E**) Schematic outlining pyrimidine biosynthesis and salvage in *S. aureus*. (**F**) Growth of *upp*::Tn *S. aureus* JE2 harboring pOS1 EV or vector containing *S. aureus upp* treated with vehicle or 100 µg/mL 4-TU.

To investigate if disruption of pyrimidine metabolism is a viable target for the development of new antimicrobials against drug-resistant *S. aureus*, the uracil analog, 4-thiouracil (4-TU), was employed. In bacteria lacking TudS, 4-TU is growth inhibitory and is incorporated into RNA (12). *S. aureus* lacks a TudS enzyme, and 4-TU has antimicrobial activity against *S. aureus* (13). To determine if 4-TU has a dose-dependent effect on *S. aureus*, the laboratory-adapted methicillin-resistant (MRSA) USA300 isolate JE2 was treated with a range of 4-TU concentrations. Indeed, 4-TU inhibited *S. aureus* growth in a dose-dependent manner (Fig. 1A). To investigate the antimicrobial activity of 4-TU against the current clinically relevant *S. aureus* strains, laboratory-adapted methicillin-susceptible strain Newman, JE2, and MRSA clinical isolate CI5296 were utilized. Treatment of all strains with 4-TU resulted in growth inhibition (Fig. 1B). In *C. difficile*, TudS enables the utilization of 4-TU as a nutrient and prevents toxicity by converting 4-TU to uracil (12). We hypothesized that heterologous expression of *tudS* in *S. aureus* would alleviate 4-TU toxicity. Indeed, constitutive expression of *tudS* improves *S. aureus* growth in the presence of 4-TU (Fig. 1C). Furthermore, consistent with *Escherichia coli* that lacks *tudS*, and a *C. difficile* mutant inactivated for *tudS* (12), cotreatment of *S. aureus* with uracil and 4-TU diminishes the antimicrobial activity of 4-TU (Fig. 1D). Collectively, these data demonstrate that 4-TU inhibits *S. aureus* growth in a dose-, nutrient-, and TudS-dependent manner.

To further interrogate the mechanism by which 4-TU inhibits *S. aureus* growth, a genetic selection in the presence of 4-TU was performed to isolate spontaneous suppressor mutant strains. Sequencing of suppressor strains revealed a nonsense mutation in the regulator of pyrimidine biosynthesis (*pyrR*), and missense and nonsense mutations in uracil phosphoribosyltransferase (*upp*) (Table 1). In *C. difficile*, mutations in *pyrR* confer resistance to 4-TU by increasing pyrimidine biosynthesis, decreasing incorporation of 4-TU into RNA, and diminishing phosphoribosyl transferase activity (12). Upp is a component of the pyrimidine salvage pathway that canonically converts uracil to uridine monophosphate (Fig. 1E). Since mutations in *upp* were observed with higher frequency, the role of *upp* in 4-TU metabolism in *S. aureus* was investigated further. Growth of *upp* suppressor strains was improved in the presence of 4-TU relative to the parental wild-type (WT) strain (Fig. S1). To validate the results of the genetic selection, a strain of JE2 harboring a transposon insertion that disrupted expression of *upp* was utilized (*upp*::Tn). Growth of *upp*::Tn was unaffected by the presence of 4-TU, and this phenotype could be complemented by expression of *upp in trans* (Fig. 1F). Furthermore, strains harboring mutations in *upp* can resist higher concentrations of 4-TU (500 µg/mL) than the concentration in which suppressor mutants were isolated (100 µg/mL) (Fig. S2). Taken together, these results suggest that Upp contributes to 4-TU-mediated growth inhibition of *S. aureus*.

**TABLE 1:** Characterization of 4-TU-resistant *S. aureus* mutants

Bacterial strain	Mutated gene	Mutation	Annotation*^a^*
*S. aureus* JE2	*upp*	C → T	**T26I** (ACT → ATT)
*S. aureus* JE2	*upp*	G → C	**A201P** (GCT → CCT)
*S. aureus* JE2	*upp*	C → A	**A64D** (GCT → GAT)
*S. aureus* JE2	*upp*	T → A	L91* (TTA → TAA)
*S. aureus* JE2	*pyrR*	A → T	R72* (AGA → TGA)

^a^ Nonsynonymous mutations are shown in bold, while nonsense mutations are indicated in lightface with an asterisk "*".

**Fig 2:**
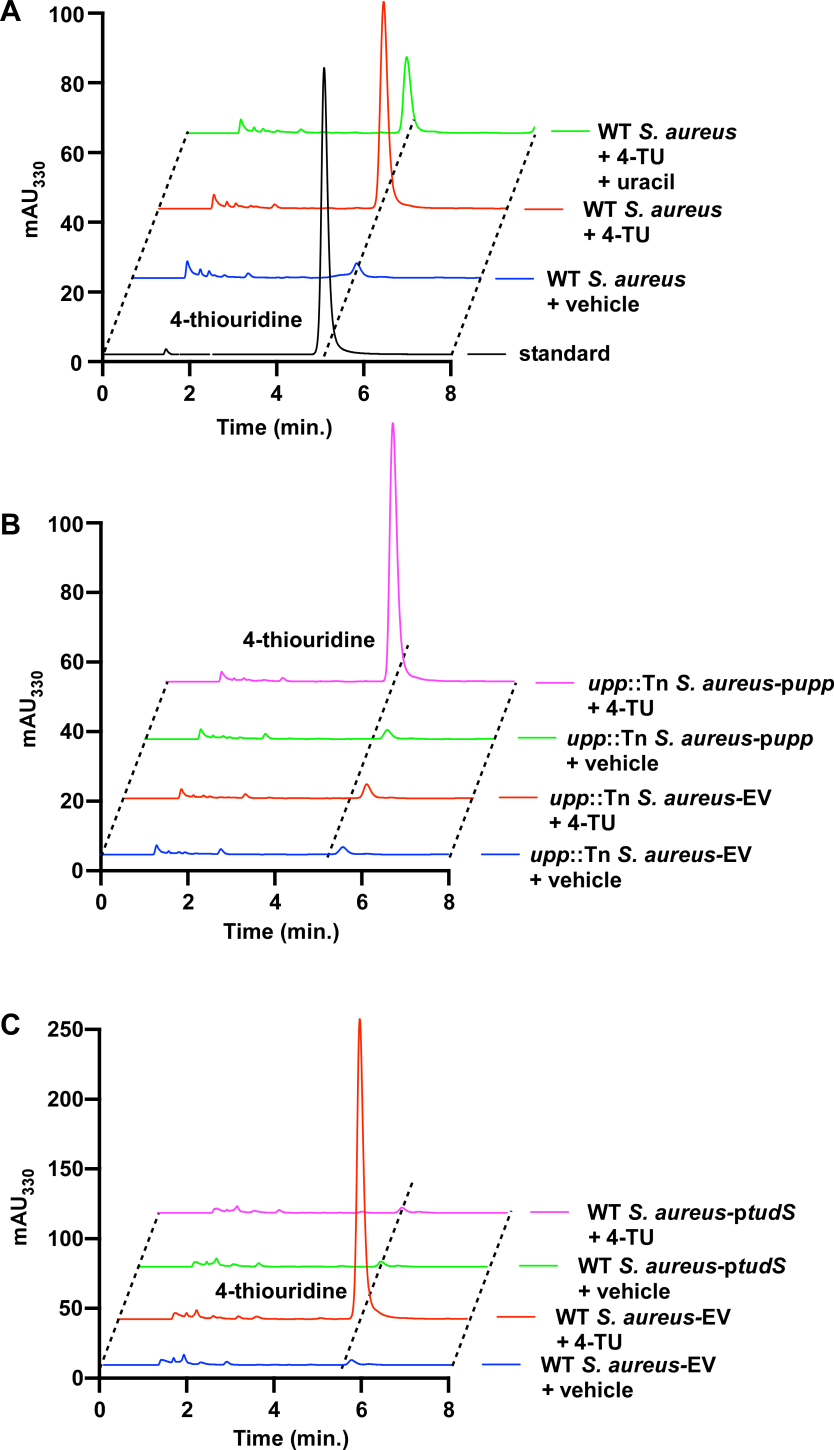
Uracil phosphoribosyltransferase facilitates 4-TU incorporation into *S. aureus* RNA. (**A**) WT *S. aureus* JE2 was treated with vehicle, 100 µg/mL 4-TU, or 100 µg/mL 4-TU and 100 µg/mL uracil in CDM, and s^4^U incorporation was determined by HPLC. (**B**) *upp*::Tn *S. aureus* JE2 harboring pOS1 empty vector (EV) or *S. aureus* upp (p*upp*) were treated with vehicle or 100 µg/mL 4-TU in CDM, and s^4^U incorporation was determined by HPLC. (**C**) WT *S. aureus* JE2 harboring pOS1 EV or *C. difficile tudS* treated with vehicle or 100 µg/mL 4-TU in CDM, and s^4^U incorporation was determined by HPLC.

Based on the structural similarity between 4-TU and uracil and the observation that cotreatment with uracil in addition to 4-TU improves growth in 4-TU (Fig. 1D), we hypothesized that 4-TU is incorporated into *S. aureus* RNA. To test this, RNA was isolated from WT *S. aureus* treated with vehicle or 4-TU, and the abundance of 4-thiouridine (s^4^U) was quantified by high-performance liquid chromatography (HPLC). WT *S. aureus* treated with 4-TU contained a peak corresponding to s^4^U, indicating incorporation into *S. aureus* RNA (Fig. 2A). To investigate if cotreatment with uracil reduces the abundance of s^4^U in RNA, WT *S. aureus* was treated with uracil and 4-TU. Indeed, cotreatment with uracil diminished s^4^U levels in RNA relative to strains treated with 4-TU alone (Fig. 2A). These data suggest that 4-TU and uracil compete for incorporation into RNA. We hypothesized that since Upp uses uracil as a substrate, and mutations in *upp* confer resistance to 4-TU in *S. aureus*, the *upp*::Tn mutant strain would have reduced s^4^U levels in RNA relative to the WT strain treated with 4-TU. Treatment of *upp*::Tn with 4-TU resulted in no detectable s^4^U in RNA, and expression of *upp in trans* in *S. aureus* in these conditions increased s^4^U levels (Fig. 2B). In *C. difficile*, *tudS* is required to prevent incorporation of 4-TU into RNA (12). Therefore, we reasoned that heterologous expression of *tudS* would prevent incorporation of 4-TU into *S. aureus* RNA. Analysis of RNA isolated from *S. aureus* expressing *tudS* lacked detectable s^4^U (Fig. 2C). Taken together, these results suggest that Upp facilitates incorporation of 4-TU into *S. aureus* RNA, and uracil and TudS diminish s^4^U levels in RNA.

The increasing emergence of drug-resistant *S. aureus* strains necessitates the development of new strategies to inhibit staphylococcal growth. Here, we show that 4-TU inhibits the growth of drug-resistant *S. aureus* strains and is incorporated into cellular RNA pools. The mechanism by which RNA containing s^4^U is toxic is not well understood, and further research to understand the consequences of s^4^U incorporation into RNA and the potential impact on translation is necessary. Furthermore, the toxicity of 4-TU is likely multifaceted, with other cellular pathways requiring uridine-containing nucleotides, such as cell wall and glycogen synthesis, potentially being affected. Our data suggest that Upp, an enzyme involved in pyrimidine salvage, facilitates incorporation of 4-TU into RNA. Upp is essential for *S. aureus* fitness in abscesses and biofilms (10, 11), suggesting that pyrimidine synthesis alone is not sufficient to meet the cellular requirements for pyrimidines in these environments and highlighting the importance of Upp-mediated salvage for survival. Collectively, these findings indicate that commandeering the pyrimidine salvage pathway may serve as a promising target to inhibit *S. aureus* growth using 4-TU, and differences between host and bacteria in the acquisition of pyrimidine nucleotides make targeting pyrimidine metabolism an attractive strategy for the treatment of infections caused by *S. aureus*.

## SUPPLEMENTAL MATERIAL

Supplemental Information spectrum.00640-25-s0001.pdf Supplemental Figures, tables, and materials and methods.

ASM does not own the copyrights to Supplemental Material that may be linked to, or accessed through, an article. The authors have granted ASM a non-exclusive, world-wide license to publish the Supplemental Material files. Please contact the corresponding author directly for reuse.
