# Supplementary material for: The nucleobase analog 4-thiouracil hijacks the pyrimidine salvage pathway to inhibit Staphylococcus aureus growth
Source: Microbiol Spectr. 2025 May 27;13(7):e00640-25. doi: 10.1128/spectrum.00640-25 (PMC12210855; doi:10.1128/spectrum.00640-25)
Supplement: Supplemental Information — Supplemental Figures, tables, and materials and methods. [file spectrum.00640-25-s0001.pdf]

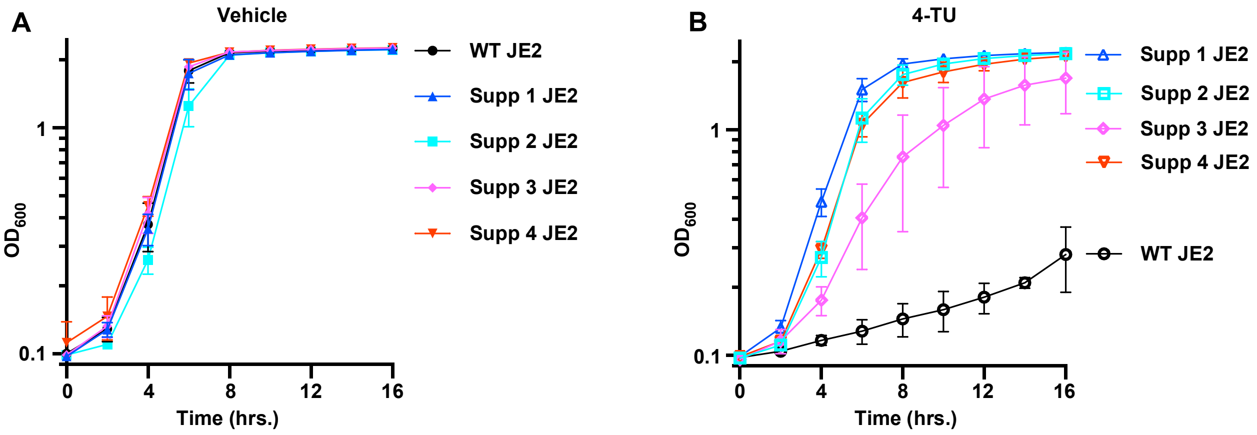

Figure S1. Growth of 4-thiouracil-resistant *S. aureus* suppressor strains. (**A**) Growth of WT *S. aureus* JE2 and spontaneous suppressor mutants treated with vehicle or (**B**) treated with 100 μg/mL 4-TU.

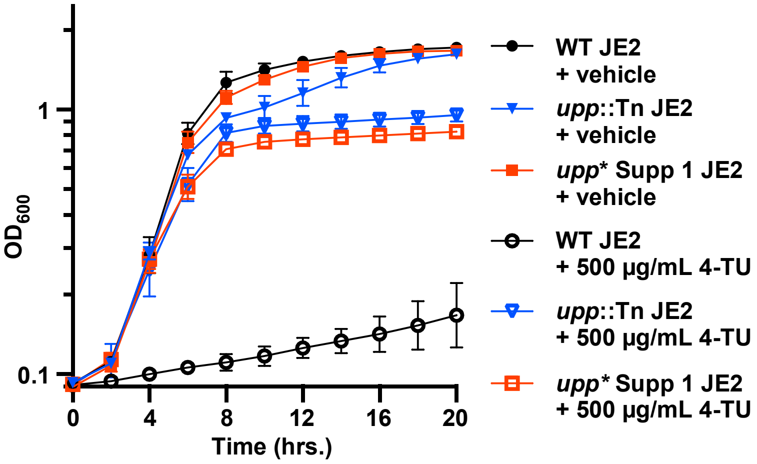

Figure S2. Mutations in *upp* confer resistance to high concentrations of 4-TU. Growth of WT, *upp*::Tn, and a representative suppressor mutant (*upp**) *S. aureus* JE2 treated with vehicle or 500 μg/mL 4-TU.

Table S1. Primers used in this study.

Primer	Primer sequence	Primer description
pOS1_tudS_F	aaatacaattgaggtgaacatATG ATATTGGTATCTGCATG	5' primer to amplify *tudS* from *C. difficile* for cloning into pOS1 expresion vector
pOS1_tudS_R	aaacactaccccttgtttggatc cCTATAAATCTTTTTCACTCTTAA TTTTTATTC	3' primer to amplify *tudS* from *C. difficile* for cloning into pOS1 expresion vector
pOS1_upp_F	aaatacaattgaggtgaacatATGAGT AAAGTACACGTTTTC	5' primer to amplify *upp* from *S. aureus* for cloning into pOS1 expresion vector
pOS1_upp_R	aaacactaccccttgtttggatc cTCAGAAATCGCTTGGTTTTTC	3' primer to amplify *upp* from *S. aureus* for cloning into pOS1 expresion vector
pOS1_seq_F	AAGAAGAGATGTAAGAGTAG GG	5' primer for sequencing pOS1
pOS1_seq_R	AATTTCACACAGGAAACAGC	3' primer for sequencing pOS1
pOS1_seq_F	AAGAAGAGATGTAAGAGTAG GG	5' primer for sequencing pOS1
pOS1_seq_R	AATTTCACACAGGAAACAGC	3' primer for sequencing pOS1
upp_SA_F	tcttgttccaaatatggtcataatc	5' primer to amplify *S. aureus* upp for Sanger Sequencing
upp_SA_R	ttaacagctgaatatcctctatatc	3' primer to amplify *S. aureus* upp for Sanger Sequencing

Table S2. Strains used in this study.

Strain	Description	Source/Reference
Escherichia coli DH5α	Wild-type laboratory stock for cloning	Lab stock
S. aureus RN4220	Wild-type restriction enzyme-deficient cloning intermediate strain	Kreiswirth et al. (1983)
S. aureus JE2	Wild-type USA300 community-acquired methicillin-resistant *S. aureus* (CA-MRSA) isolate	Fey et al. (2013)
S. aureus Newman	Wild-type methicillin-sensitive *S. aureus* (MSSA) clinical isolate	Duthie et al. (1952)
S. aureus CI5296	Wild-type methicillin-resistant *S. aureus* (MRSA) isolated from the blood of a patient with endocarditis	Freiberg et al. (2024)
upp::Tn *S. aureus* JE2	*NE21; SAUSA300_2066*::Tn	Fey et al. (2013)
Escherichia coli DH5α pOS1	Cloning strain with pOS1 containing the *lgt* promoter	Schneewind et al. (1992)
S. aureus JE2 pOS1	Wild-type JE2 with pOS1 empty vector	This study

S. aureus JE2 pOS1-*tudS*	Wild-type JE2 with pOS1 harboring *tudS* from *C. difficile* downstream of the *lgt* promoter	This study
upp::Tn *S. aureus* JE2 pOS1	*SAUSA300_2066*::Tn with pOS1 empty vector	This study
upp::Tn *S. aureus* JE2 pOS1-*upp*	*SAUSA300_2066*::Tn with pOS1 harboring upp from *S. aureus* downstream of the lgt promoter	This study

Materials and Methods

Materials. *S. aureus* JE2 and NE21 were provided by the Network on Antimicrobial Resistance in *Staphylococcus aureus* (NARSA) for distribution by BEI Resources, NIAID, NIH: Nebraska Transposon Mutant Library (NTML) Genetic Toolbox, NR-48850. *S. aureus* Newman was a generous gift from Dr. Olaf Schneewind. *S. aureus* CI 5296 is a clinical methicillin-resistant *S. aureus* isolate that was obtained from the Vanderbilt University Medical Center (VUMC) clinical microbiology laboratory with approval from the VUMC Institutional Review Board. CI 5296 was initially isolated from the blood culture of a patient with endocarditis. Strain *upp*::Tn was constructed by phage transduction of *upp*::*erm* from NE21 into JE2 via φ85. The *upp*::Tn complemented strain was constructed by cloning *upp* from *S. aureus* downstream of the *lgt* promoter in pOS1. The *tudS* expressing strain was constructed by cloning *tudS* from *C. difficile* downstream of the *lgt* promoter in pOS1. Constructs were assembled using NEB HiFi DNA Assembly. The resulting plasmids were transformed into RN4220 and then into WT or *upp*::Tn JE2. Correct construction of these strains was confirmed by PCR and by whole genome sequencing. *S. aureus* strains were grown on tryptic soy agar (TSA), tryptic soy broth (TSB) (Becton-Dickinson), or a modified chemically defined media for *S. aureus* culture using casamino acids (CDM) (1). Chemicals were obtained from Sigma and plasticware was from USA Scientific

or Corning. Data were plotted in Prism 10 (GraphPad) and figures were generated in Canvas X Draw 7 (Canvas GFX).

***S. aureus* overnight cultures.** WT *S. aureus* strains were streaked on TSA, *upp*::Tn was streaked on TSA containing 10 µg/mL erythromycin, and strains harboring pOS1 were streaked on TSA containing 10 µg/mL chloramphenicol and incubated at 37ºC for 16 h. Single colonies were inoculated into 5 mL TSB, TSB with 10 µg/mL erythromycin, or TSB with 10 µg/mL chloramphenicol and were incubated at 37ºC with orbital shaking at 180 rpm and a 45º angle for 16 h in an Innova44 shaking incubator (Eppendorf).

***S. aureus* kinetic growth curves.** *S. aureus* overnight cultures grown in triplicate were diluted 1:100 into CDM, with antibiotics where appropriate, and incubated at 37ºC with orbital shaking at a 45º angle for 2 h. Bacterial cultures were diluted 1:50 into a 96-well plate containing 200 µL of CDM with the conditions to be tested. For Figure 1, bacteria were incubated with vehicle (dimethyl sulfoxide, Sigma), 100 µg/mL 4-thiouracil, or 100 µg/mL 4-thiouracil with 100 µg/mL uracil, with antibiotics where appropriate. For Figure S1 bacteria were incubated with vehicle or 100 µg/mL 4-thiouracil. All growth curves were performed at 37ºC with linear shaking at 567 cpm (3 mm) with optical density measurements at 600 nm (OD_{600}) every 30 min on an EPOCH 2 plate reader (Bio Tek, Winooski, VT).

RNA isolation. *S. aureus* overnight cultures grown in triplicate were diluted 1:100 into CDM, with antibiotics where appropriate, and incubated at 37ºC with orbital shaking at a 45º angle to an OD_{600} of 0.3. Cultures were treated with either vehicle (dimethyl sulfoxide), 100 µg/mL 4-thiouracil, or 100 µg/mL 4-thiouracil with 100 µg/mL uracil and incubated at 37ºC with orbital shaking at a 45º angle for 1 h. Following 1 h of treatment, bacteria were centrifuged and resuspended in 1 mL of TRIzol Reagent (Invitrogen), then stored at -80°C until time of RNA

isolation. For RNA isolation, resuspended bacteria were homogenized in a bead beater with Lysing Matrix B beads (MP Biomedical) at a speed of 6 m/s for 45 seconds. Homogenized suspensions were centrifuged and the upper phase was collected and mixed with 200 μL chloroform (Acros Organics). Samples were incubated at room temperature for 2 min, centrifuged at 4°C for 15 min, and 400 μL of the upper aqueous phase was collected and mixed with an equal volume of 100% ethanol. RNA was then extracted using a RNeasy RNA extraction kit (Qiagen) according to the manufacturer's instructions. RNA was eluted from purification columns using 53 µL UltraPure water (Invitrogen). DNA contamination was removed using the TURBO DNase kit (Invitrogen) according to the manufacturer's instructions and RNA was stored at -80°C.

s^4U incorporation assay. Bacterial ribonucleosides were isolated as previously described (2). Nucleosides derived from RNA and standards were analyzed on an Agilent 1260 Infinity II system. Analytes were separated by isocratic high-performance liquid chromatography (HPLC) on a Supelco Ascentis Express C_{18} column (25 cm x 2.1 mm, 5 μm) with a Phenomenex SecurityGuard C_{18} cartridge (3.2 x 8 mm) at a flow rate of 0.4 mL/min using 0.1% trifluoroacetic acid in water as the mobile phase for 20 min. 4-thiouridine was detected at 330 nm and the retention time was confirmed using a purified standard (Sigma).

Isolation of 4-TU resistant mutants. Overnight cultures of WT *S. aureus* JE2 started from independent colonies were subcultured 1:100 in CDM for 2 h and diluted 1:50 into a 96 well plate containing vehicle or 100 μg/mL 4-TU. Growth was monitored in an Epoch 2 microplate reader (Biotek) and suppressor from individual wells demonstrating growth in 4-TU were isolated on TSA. Genomic DNA (gDNA) was extracted using the Qiagen DNeasy Blood and Tissue kit according to the manufacturer's instructions with a lysostaphin pretreatment step. Mutations in

upp were identified by PCR amplification and Sanger sequencing of purified gDNA from suppressor strains using the primers listed in **Table S1**. The mutation in *pyrR* was identified by whole genome sequencing performed by SeqCenter (Pittsburgh, PA) using 150-bp paired end reads on Illumina NextSeq 2000. Single nucleotide polymorphisms (SNPs) were identified in *S. aureus* accession CP020619 using the breseq pipeline (3). Whole genome sequencing data are available in the National Center for Biotechnology Information (NCBI) sequence read archive (SRA) under BioProject: PRJNA1246203.

References

1. Vitko NP, Richardson AR. 2013. Laboratory maintenance of methicillin-resistant *Staphylococcus aureus* (MRSA). Curr Protoc Microbiol Chapter 9:Unit 9C.2.
2. Munneke MJ, Yuan Y, Preisner EC, Shelton CD, Carroll DT, Kirchoff NS, Dickson KP, Cantu JO, Douglass MV, Calcutt MW, Gibson-Corley KN, Nicholson MR, Byndloss MX, Britton RA, de Crécy-Lagard V, Skaar EP. 2025. A thiouracil desulfurase protects *Clostridioides difficile* RNA from 4-thiouracil incorporation, providing a competitive advantage in the gut. Cell Host Microbe doi:10.1016/j.chom.2025.03.001.
3. Deatherage DE, Barrick JE. 2014. Identification of mutations in laboratory-evolved microbes from next-generation sequencing data using breseq. Methods Mol Biol 1151:165-88.
